# Epidural Blood Patching in Spontaneous Intracranial Hypotension—Do we Really Seal the Leak?

**DOI:** 10.1007/s00062-022-01205-7

**Published:** 2022-08-26

**Authors:** Eike I. Piechowiak, Benjamin Aeschimann, Levin Häni, Johannes Kaesmacher, Pasquale Mordasini, Christopher Marvin Jesse, Christoph J. Schankin, Andreas Raabe, Ralph T. Schär, Jan Gralla, Jürgen Beck, Tomas Dobrocky

**Affiliations:** 1 Department of Diagnostic and Interventional Neuroradiology, Inselspital, Bern University Hospital, University of Bern, Freiburgstr. 8, 3010 Bern, Switzerland; 2 Department of Neurosurgery, Inselspital, Bern University Hospital, and University of Bern, Bern, Switzerland; 3 Department of Neurology, Inselspital, Bern University Hospital and University of Bern, Bern, Switzerland; 4 Department of Neurosurgery, Medical Center—University of Freiburg, Freiburg, Germany

**Keywords:** Spontaneous intracranial hypotension, Cerebrospinal fluid leak, Epidural blood patch

## Abstract

**Purpose:**

Epidural blood patch (EBP) is a minimally invasive treatment for spontaneous intracranial hypotension (SIH). Follow-up after EBP primarily relies on clinical presentation and data demonstrating successful sealing of the underlying spinal cerebrospinal fluid (CSF) leak are lacking. Our aim was to evaluate the rate of successfully sealed spinal CSF leaks in SIH patients after non-targeted EBP.

**Methods:**

Patients with SIH and a confirmed spinal CSF leak who had been treated with non-targeted EBP were retrospectively analyzed. Primary outcome was persistence of CSF leak on spine MRI or intraoperatively. Secondary outcome was change in clinical symptoms after EBP.

**Results:**

In this study 51 SIH patients (mean age, 47 ± 13 years; 33/51, 65% female) treated with non-targeted EBP (mean, 1.3 EBPs per person; range, 1–4) were analyzed. Overall, 36/51 (71%) patients had a persistent spinal CSF leak after EBP on postinterventional imaging and/or intraoperatively. In a best-case scenario accounting for missing data, the success rate of sealing a spinal CSF leak with an EBP was 29%. Complete or substantial symptom improvement in the short term was reported in 45/51 (88%), and in the long term in 17/51 (33%) patients.

**Conclusion:**

Non-targeted EBP is an effective symptomatic treatment providing short-term relief in a substantial number of SIH patients; however, successful sealing of the underlying spinal CSF leak by EBP is rare, which might explain the high rate of delayed symptom recurrence. The potentially irreversible and severe morbidity associated with long-standing intracranial hypotension supports permanent closure of the leak.

## Introduction

Spontaneous intracranial hypotension (SIH) is an important cause of secondary, predominantly orthostatic, headache [1, 2]. The positional headache characteristics may dwindle over time, resulting in a daily chronic headache [3, 4], or paradoxical headache patterns, with worsening in the recumbent position [1, 3]. Various additional symptoms, including neck pain, neck stiffness, nausea, vomiting, tinnitus, diplopia, and decreased level of consciousness have been described [1, 3–5]. Three etiologies, first described by Schievink et al. in 2016, that may lead to spinal CSF loss include ventral dural tear (type 1), leaking nerve root diverticulum (type 2) or a CSF-venous fistula (CVF; type 3) [4, 6–9].

Epidural blood patch (EBP) therapy is the second line treatment when conservative measures like bed rest, hydration and caffeine fail [1, 3–5, 8]. Despite almost half a century of experience with EBP for SIH treatment, no randomized controlled studies have been performed. Therefore, the mode of action of this therapy and its long-term efficacy are still unclear.

Variations of the treatment approach contribute to the inconsistent results. These include the amount (small or large volume), type of product injected (autologous blood or fibrin), means of delivery (targeted or non-targeted), and navigation (imaging-guided or blind) of the EBP. Furthermore, monitoring in SIH patients varies widely according to local practice and reported effect parameters are not uniform, often subjective, and include headache intensity, headache frequency, and headache duration as well as the subjective improvement of symptoms. Several studies merely report on complete orthostatic headache remission, which might not reflect the complex clinical presentation in SIH patients with diverse symptoms.

Although considered crucial during the diagnostic work-up, spine imaging is often neglected during follow-up and the imaging proof of successful CSF leak sealing is rarely sought [10, 11].

Moreover, the correlation between clinical evolution and spine imaging findings after EBP has not been reported.

The goal of our study was primarily to evaluate the rate of a successful spinal CSF leak closure and to correlate the clinical response after non-targeted EBP in SIH patients with a proven spinal CSF leak.

## Material and Methods

The study protocol was approved by the local ethics committee and the requirement for informed consent was waived due to the retrospective nature of this single-center study.

### Study Design

Medical records of consecutive SIH patients with a myelographically confirmed CSF leak, treated with a non-targeted EBP at our institute between January 2012 and November 2019 were reviewed. Exclusion criteria were a lack of a CSF leak on spinal imaging or if the patients have had previous spinal interventions like surgery.

Age, sex, symptoms (typical or atypical), number of EBPs performed, the volume injected into the epidural space, imaging studies, and surgical reports after a failed EBP were retrieved from the local clinical database and the picture archiving and communications system. Typical symptoms of SIH were defined as orthostatic headache and additional symptoms associated with SIH, such as neck pain and tinnitus. Atypical symptoms were defined as nonpostural headache patterns, absence of headache and dominance of other associated symptoms (e.g., nausea, diplopia, hearing loss).

### Clinical Presentation and Standard of Care

**Fig. 1:**
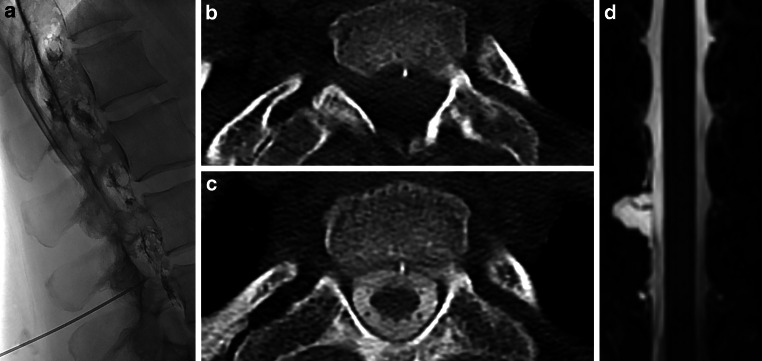
**a** Distribution of blood and contrast agent in the epidural space during epidural blood patch. **b** Discogenic microspur on an axial CT image. **c** Same level after contrast agent injection. **d** Coronal T2-weighted MRI showing a ruptured nerve root cyst and adjacent epidural CSF collection

All patients fulfilled the SIH criteria according to the International Classification of Headache Disorder (ICHD [12]) and demonstrated a spinal longitudinal epidural CSF collection (SLEC) on multimodal spine imaging including MRI, conventional dynamic myelography, or dynamic CT myelography (Fig. 1; [7]).

According to our clinical standard, a non-targeted EBP was performed when conservative measures failed to provide long-term relief. Repeated EBP had been discussed interdisciplinarily with the referring neurosurgeon or neurologist, typically when clinical symptoms improved but did not completely resolve after the first EBP.

**Fig. 2:**
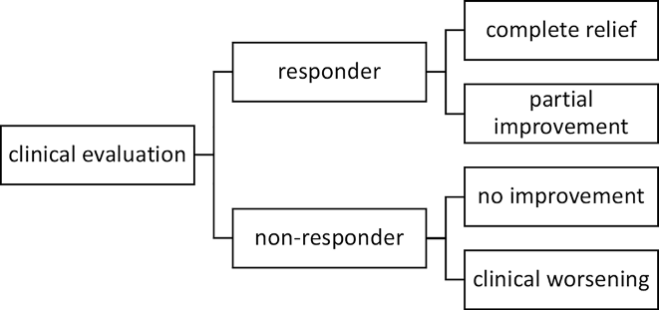
Flow chart showing the clinical evaluation

The effect of the EBP and the extent of the postinterventional change in symptoms was evaluated from the clinical assessment reports of the attending physicians during a follow-up visit. Complete relief of symptoms was defined as resolution of all symptoms associated with SIH. Partial improvement was defined as persistent but attenuated symptoms. Patients whose symptoms completely resolved or who reported a partial improvement were referred to as responders. Non-responders were patients with unchanged symptoms or clinical worsening (Fig. 2). The clinical outcome was summarized as short-term outcome, correlating the clinical status of the patients within the 7 days following the EBP, with long-term outcome defined as the patients’ health status after the third postinterventional week.

Further steps after clinical or imaging follow-up were decided for each patient individually and included additional EBP, surgical intervention or further conservative treatment.

The anonymized data were edited in REDCap (Research Electronic Data Capture) and evaluated with descriptive statistics.

### Imaging

Brain MRI with intravenous gadolinium was acquired on a 1.5-Tesla or 3‑Tesla scanner. Spine MRI was acquired on a 1.5-Tesla scanner (Aera; Siemens, Erlangen, Germany) using a 20-element head/neck coil and a 32-element table spine coil. As previously described, the unenhanced spine MRI protocol included 3 sagittal T2-weighted spin-echo blocks (TE 110, TR 4350, time of acquisition: 03:33 min:sec, flip angle: 150°) and 4 sagittal, isotropic 3D heavily T2-weighted turbo spin-echo sequences with fat saturation (TE 180, TR 1400, time of acquisition: 04:33 min:sec, flip angle: 150°) including the entire neurocranium [13].

Conventional or dynamic CT myelography was performed during intrathecal injection of non-ionic contrast agent to localize the exact site of the CSF leak. This technique has been previously described [8, 14].

Briefly, for conventional dynamic myelography the mobile table was tilted into the Trendelenburg position, with the x‑ray detector following the leading edge of the intrathecal contrast. agent The goal was to identify the level where contrast started spilling from the intrathecal into the epidural compartment. The patient was then transferred to the CT imaging suite, and postmyelography CT (PMCT) was performed (Somatom Definition Edge, Siemens, Erlangen, Germany) to identify the underlying pathology (e.g., disc protrusion with an osseous spur) [13].

#### Image Analysis

The MRI studies of all patients were assessed by one board-certified neuroradiologist (E.P.) with 12 years of experience, and a medical student with a special interest and training in SIH (B.A.). Both readers were blinded to clinical presentation, other imaging studies, or any details of EBP therapy. Studies were reviewed on a PACS station.

The readers were instructed to carry out their assessment and report the results in a standardized spreadsheet, and a short training module was provided before beginning the image interpretation. Baseline and follow-up spine MRI studies were randomly mixed. Reading was performed using the T2-weighted spin-echo blocks and/or isotropic 3D heavily T2-weighted turbo spin-echo sequences with fat saturation. The presence of SLEC was recorded as was the type of leak, together with possible reasons, such as a microspur or ruptured nerve root cysts (Fig. 1).

### Epidural Blood Patch Technique

The non-targeted EBP procedure was performed on a monoplane high-resolution angiographic system (Artis zee multipurpose, Siemens, Erlangen, Germany) equipped with a flat panel detector (30 × 40 cm) with the patient in a sitting or lateral decubitus position. Under fluoroscopic guidance a 17-gauge epidural needle (Espocan®, B. Braun, Melsungen, Germany) was slowly advanced into the epidural space in the lumbar spine (L2/3, L3/4 or L4/5) with continuous pressure applied to the syringe plunger, known as the loss-of-resistance technique. The epidural needle was angled cranially to promote more rostral flow of the injected blood. The position of the needle tip in the epidural compartment was confirmed with a small amount of ionic contrast medium (Iopamiro 300, Iopamidol, Bracco, Cadempino, Switzerland). If the puncture was performed with the patient in the sitting position, the table was then tilted into the horizontal position and the sterile autologous blood was slowly injected. The added contrast agent demonstrated that the blood spread cranially along the spinal canal to the thoracic spine. The volume of injected blood was adapted to each individual and based on the patient feedback. The injection was paused when patients reported discomfort in the lower back or radiating pain and cramps in their buttocks and legs and resumed when these symptoms subsided. The injection was stopped, and the needle removed if slight discomfort persisted for an extended period. The patients were asked to remain in slight Trendelenburg position for 6 h to maximize distribution of the injected blood along the spine [15].

## Results

In total, 51 patients (65% women and 35% men) were included in the final study cohort (mean age 47 years; range 13–74 years). Of these, 46/51 (90%) patients presented typical SIH symptoms, whereas 5/51 (10%) had atypical symptoms. At baseline, spine MRI and conventional dynamic myelography with PMCT were obtained for 44/51 (86%) patients, whereas only spine MRI was available for the remaining 7/51 (14%) patients. All patients demonstrated a SLEC+ leak. The exact level of leakage was identified in 73% (37/51) of patients, and 95% (35/37) of these leaks were located in the thoracic spine or cervicothoracic junction (Table 1).

**Table 1:** Demographic data, clinical and imaging information, and epidural blood patch (EBP) characteristics

	Spontaneous intracranial hypotension (*n* = 51)	%
*Age*		
Mean (years)	46.6	–
Range (years)	12.7–73.5	–
Female	33	64.7
*SIH symptoms*		
Typical^a^ symptoms	46	90.2
Atypical^b^ symptoms	5	9.8
*Spinal imaging*		
Spinal MRI + myelography with PMCT	44	86.3
Spinal MRI	7	13.7
*Extradural CSF detected*	51	100
Leak localized	37	72.5
Thoracic or cervicothoracic junction	35	94.6
Leak level not specified	14	27.5
*Number of EBPs*	67	100
1	38	74.5
2	11	21.6
3	1	2.0
4	1	2.0
*Volume of EBP*		
Mean (ml)	25.1	–
Range (ml)	10.0–53.0	–

^a^Orthostatic headache, neck pain, hearing disturbances, etc.

^b^Non-postural headache patterns or absence of headache

### Primary Outcome—Successful CSF Leak Sealing

**Fig. 3:**
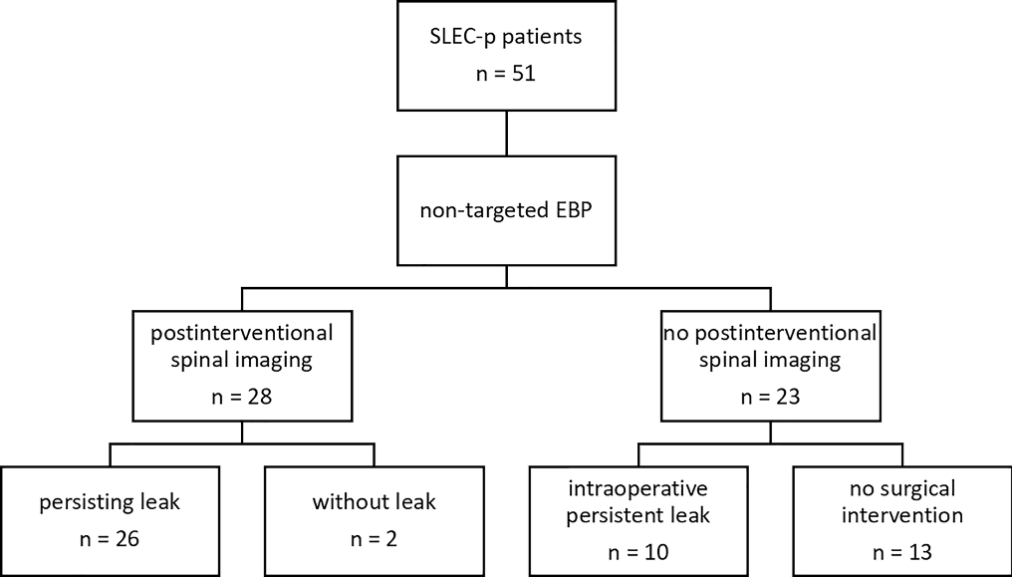
Flow chart after EBP with imaging or surgical evaluation at follow-up

Owing to persisting symptoms in 28/51 (55%) patients after EBP therapy, spinal images (spine MRI and/or CT myelography) were acquired (Fig. 3). Mean time between EBP and imaging was 14 weeks (range 1–112 weeks). In 26/28 (93%) of these patients a SLEC+ was demonstrated after the last EBP. Two patients (7%) did not show any SLEC on post-EBP imaging. One had a ruptured nerve root cyst, diagnosed by dynamic myelography, and the MRI was acquired after the fourth EBP, and the second had multiple nerve root cysts and there was no evidence of SLEC after the second EBP. In addition, in 10/51 (20%) patients, EBP failed to provide clinical relief, and they were referred for surgery without additional imaging. In all these patients, a dural breach with an epidural CSF collection was present intraoperatively. In the remaining 13/51 (25%) patients, no postinterventional spine MRI and/or CT myelography was available and no surgery was performed.

In summary, a persistent spinal CSF leak was present after EBP in 36/51 patients (71%). Even in the best-case scenario accounting for missing data, if the leak had been successfully sealed in all patients with missing post-EBP spine MRI who had not undergone surgery, this would result in a maximum success rate of 29% (15/51 patients; 2 with a sealed leak verified on imaging (1 and 3 weeks after EBP) + 13 with no follow-up imaging).

### Secondary Outcome—Clinical Findings

The short-term outcome after the first postinterventional week was available for all enrolled patients (51/51, 100%). Overall, 45/51 (88%) patients responded to the first EBP in the short-term and only 12% (6/51) showed no improvement of the clinical condition (Table 2). None of the patients reported symptom worsening. If symptoms recurred after the first EBP, additional EBPs were performed according to the patient’s wishes and the decision of a multidisciplinary SIH board. Therefore, a total of 2, 3 or 4 EBPs were performed in 11 (22%), 1 (2%), and 1 (2%) patients, respectively (Table 1). Data for the long-term outcome were available for 47/51 (92%) patients with a mean follow-up period of 6.5 months (range, 1–50 months). Overall, 17/47 (36%) patients responded to the EBP in the long-term and 30/51 (59%) patients remained non-responders in the long-term. The main side effect reported was mostly low back pain for 1–4 days as well as headache and tinnitus in the first 24 h after treatment. No complications or long-term side effects were reported.

**Table 2:** Primary and secondary outcome

	Spontaneous intracranial hypotension (*n* = 51)	%
*Short-term outcome*	51	100
No improvement	6	11.8
Partial improvement	11	21.6
Complete relief	34	66.7
Clinical worsening	0	0
*Long-term outcome*	51	100
No improvement	30	58.8
Partial improvement	6	11.7
Complete relief	11	21.6
After 2nd or 3rd EBP	2	3.9
Lost to follow-up	4	7.8
*Postinterventional spinal imaging*	28	54.9
Persisting CSF leak	26	50.9
No CSF leak	2	3.9
Surgical intervention	27	52.9

*CSF* cerebrospinal fluid, *EBP* epidural blood patch

In total, 27/47 (57%) patients subsequently underwent surgical closure of the underlying CSF leak because of persisting symptoms after EBP. Of the 51 patients 4 (8%) remained non-responders but did not undergo surgery.

## Discussion

First, our data show that orthostatic symptoms in many patients decrease in the early follow-up period after EBP therapy. Second, this effect seems temporary, and a significant number of patients reported symptom recurrences at long-term follow-up. Third, after untargeted EBP, most SIH patients demonstrated evidence of a persisting CSF leak on spinal imaging (MRI) or intraoperatively.

Response rates after 1 EBP reported in the literature ranged between 36% and 88% [10, 11, 16–19]. Some studies used the targeted, some the untargeted EBP technique, each with good success rates, while other studies have seen better response rates after targeted EBP therapy. The reasons for the striking differences in EBP efficacy are manifold. First, the approach to epidural blood patching varies substantially between studies [10, 11, 18–20]. Second, some studies do not clearly distinguish between SIH and intracranial hypotension after lumbar puncture; the latter is likely to have a more favorable prognosis for spontaneous healing. Third, treatment monitoring in SIH patients has not been standardized and varies widely between institutions. Most studies base the response rates after EBP on clinical symptoms (i.e., headache intensity) and not on imaging proof of SLEC resolution. A standardized and objective tool for treatment follow-up is lacking.

**Fig. 4:**
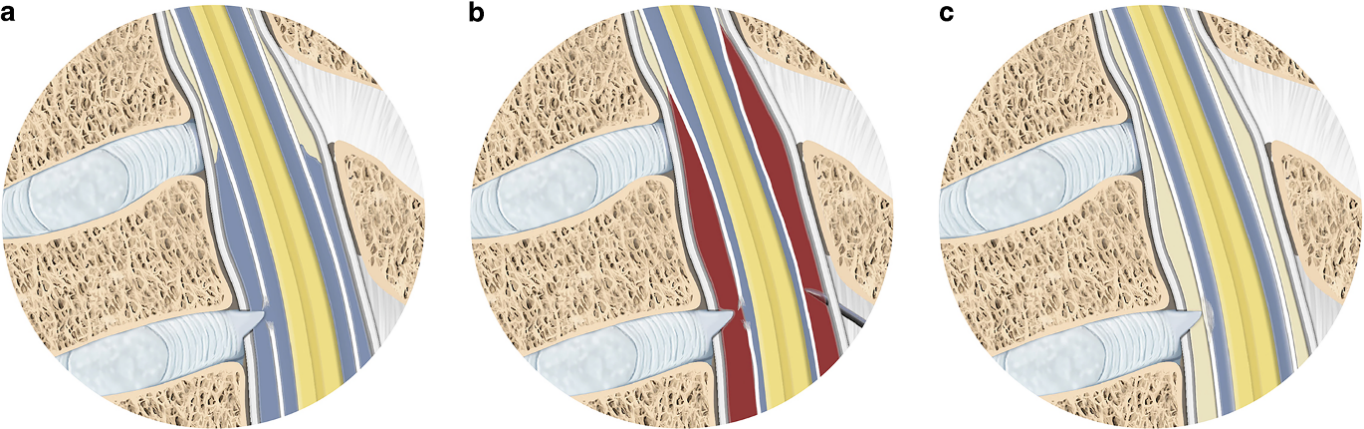
Illustration of the mechanism of epidural blood patching. **a** Spinal longitudinal CSF collection due to a discogenic microspur penetrating the ventral dura. **b** A needle navigated into the dorsal epidural space. After epidural injection of autologous blood, local compression of the thecal sac is demonstrated, leading to a decreased compliance of the CSF compartment. **c** Occlusion of the dural breach due to fibrous transformation of the coagulated blood inside the leak

The first theory of how EBP works relates to a compression of the thecal sac as a result of blood being injected in the epidural space This implies a decreased thecal sac compliance, which results in a reduced CSF shift from the cranial to the spinal compartment after changing from the recumbent to the upright position (Fig. 4b; [1, 5, 21, 22]).

This mechanism most likely accounts for the immediate improvement seen in the early follow-up period. However, resorption of injected blood within the epidural compartment over time results in decreasing compression of the thecal sac, leading again to an increased compliance and may thus explain the decreasing effectiveness seen at long-term follow-up [22, 23].

The second theory, namely the “patch effect”, is that blood injected into the epidural compartment coagulates and forms a plug around the dural breach and thus prevents further leakage of CSF into the epidural space (Fig. 4c; [1, 5]). Ventral dural leaks (leak type 1) are commonly associated with microspurs (calcified disc protrusions or endplate osteophytes) [6]. Intraoperatively, these present as sharp, almost blade-like, protrusions penetrating the dura. The idea that the resilience of a coagulated blood clot provides better stability than the dural covering itself and can lead to a permanent sealing of the dural breach seems questionable (Fig. 4c).

In our study cohort only two patients with a spinal nerve root cyst (leak type 2) demonstrated resolution of SLEC on follow-up imaging. No patient with a ventral microspur showed resolution of epidural CSF collection after EBP. Thus, according to our results, even when the best-case scenario is applied to account for missing data, the success rate of untargeted EBP sealing a spinal CSF leak remains low, with a maximum of 29%, calling into question whether the dural breach can be permanently sealed by the proposed mechanism of action.

Based on the current study a temporary closure of the leak shortly after the blood patch with subsequent rupture cannot be ruled out. This would require a prospective study with a standardized follow-up with multiple MRI examinations. Therefore, we focused on permanent closure and this should be completed after resorption of the blood.

### Permanent Sealing of a Spinal CSF Leak?

Even though untargeted EBP seems to be an effective symptomatic therapy in the acute stage, it rarely seals the underlying dural breach permanently. Thus, even if clinical improvement occurs in the early follow-up period, persisting CSF loss may perpetuate the disease and potentially result in serious long-term morbidity. This raises the question of whether untargeted EBP therapy is a causal or merely a symptomatic therapy. Should the permanent sealing of an underlying CSF leak in SIH patients be the main goal?

To maintain a stable intracranial volume, which is essential in a rigid structure such as the skull, CSF loss is compensated by a volume increase in other intracranial compartments [5]. Intracranial CSF depletion results in typical brain imaging findings such as dural enhancement, engorgement of venous sinuses, brain sagging, and subdural hematoma [1]. Consequently, CSF loss may result in serious conditions including subdural hematomas requiring decompressive craniotomy, non-convulsive status epilepticus, cognitive decline (frontotemporal brain sagging syndrome) or even coma [1, 24–28]. Several studies have reported on patients with extensive superficial siderosis linked to a CSF leak of long duration [5, 29]. The proposed mechanism for this is the bleeding from small bridging veins, which have become stretched due to brain sagging. Thus, maintaining a CSF equilibrium appears to be important for preventing morbidity and preserving a good quality of life and may thus justify a more aggressive treatment approach.

A recent study reported that symptom duration before surgical closure of a CSF leak was the most important predictor of good outcome (improvement of orthostatic headache intensity). The authors concluded that treatment should be initiated within 12 weeks after symptom onset [30].

Our study cohort is comparable to those in previously published studies in terms of age, sex and CSF leak location (95% of leaks at the cervicothoracic or thoracic level) [1, 10, 16–18]. Likewise, the EBP technique used during the study period is equivalent to that used in other centers. Usually, 10–20 ml of autologous blood was applied with the targeted or untargeted EBP method in the mentioned studies. However, the success rate of the untargeted EBP therapy in our study, defined as resolution of SLEC on follow-up imaging or intraoperatively and long-term clinical improvement, is lower than in most other studies.

There are many possible explanations for this difference. First, previous studies relied on clinical parameters only and not on imaging during follow-up. Proof of successful sealing of the dural breach was not required for evaluation of the EBP effect. Second, the inclusion criteria in our study were strict since only SIH patients with a proven CSF leak (on spinal imaging) were considered. Third, a change in headache phenotype or intensity after EBP therapy is considered as treatment success, which might not truly reflect the complex clinical presentation of SIH patients who often exhibit multiple symptoms. Fourth, the clinical presentation after EBP therapy does not always correlate with imaging findings [9]. In some of our patients, symptoms resolved after EBP even though the CSF leak was still present. Conversely, some patients reported persistent symptoms even though imaging showed no epidural CSF leak in spine MRI. This highlights the discrepancy between the clinical presentation and objective imaging proof of epidural CSF leak in SIH patients after EBP. The clinical presentation after treatment can pose diagnostic difficulties, may be subjective, and does not prove that the underlying spinal CSF leak is successfully sealed. Thus, evaluating the success of EBP therapy based on the clinical status without spinal imaging is insufficient.

In conclusion, our retrospective data analysis showed that in most patients with SIH due to CSF leak untargeted EBP alone is unlikely to improve patients clinically or seal spinal CSF leaks. This was confirmed on spinal imaging and/or intraoperatively and was also correlated with the clinical status of the patients. Thus, by performing EBP, which is often a symptomatic therapy with a low rate of successful sealing of the underlying dural breach, we might delay or even prevent adequate causal therapy and thus fail to avoid worsening of the disease. According to our data, patients with SIH due to radiologically proven CSF leaks should be actively investigated for the source of the leak and undergo subsequent early surgical intervention. In addition, the costs and the risks of the EBP therapy may not be in proportion to its limited effectiveness of the untargeted EBP therapy.

### Strengths and Limitations

The major strength of this study is the carefully selected study cohort that included only SIH patients with a proven spinal CSF leak. Second, EBP procedures were carried out in a standardized fashion: image-guided and non-targeted, requiring confirmation of the correct position of the needle tip with contrast agent. There are some limitations inherent to the retrospective nature of the study, accounting for missing data and the small study size. Furthermore, no patients with a CVF were included, since EBP therapy has been previously demonstrated to provide poor results in patients with this condition [31].

## Conclusion

Untargeted EBP is an effective symptomatic therapy providing short-term relief in a substantial number of SIH patients with proven spinal CSF leak. However, it rarely permanently seals that spinal CSF leak, which in turn might explain the high rate of delayed symptom recurrence. Even though successful sealing of the underlying spinal CSF leak is currently not a requirement, the potentially irreversible and severe morbidity associated with long-standing intracranial hypotension might justify doing so. Therefore, randomized controlled trials on different therapeutic approaches in patients with SIH are warranted.

## Abbreviations

CSF: Cerebrospinal fluid
CVF: CSF-venous fistula
EBP: Epidural blood patch
ICHD: International Classification of Headache Disorder
MRI: Magnetic resonance imaging
PMCT: Postmyelography CT
SIH: Spontaneous intracranial hypotension
SLEC+/−: Positive or negative for spinal longitudinal extradural CSF collection
